# Pathology Laboratory Surveillance in the Australian Collaboration for Coordinated Enhanced Sentinel Surveillance of Sexually Transmitted Infections and Blood-Borne Viruses: Protocol for a Cohort Study

**DOI:** 10.2196/13625

**Published:** 2019-08-08

**Authors:** Caroline van Gemert, Rebecca Guy, Mark Stoove, Wayne Dimech, Carol El-Hayek, Jason Asselin, Clarissa Moreira, Long Nguyen, Denton Callander, Douglas Boyle, Basil Donovan, Margaret Hellard

**Affiliations:** 1 Burnet Institute Melbourne Australia; 2 Department of Epidemiology and Preventative Medicine Monash University Melbourne Australia; 3 Melbourne School of Population and Global Health The University of Melbourne Parkville Australia; 4 The Kirby Institute University of New South Wales Sydney Australia; 5 NRL Melbourne Australia; 6 Department of General Practice The University of Melbourne Parkville Australia; 7 Doherty Institute Melbourne Australia; 8 Department of Infectious Diseases The Alfred Hospital Melbourne Australia

**Keywords:** hepatitis, HIV, sexually transmitted diseases, laboratories, epidemiology, Australia

## Abstract

**Background:**

Passive surveillance is the principal method of sexually transmitted infection (STI) and blood-borne virus (BBV) surveillance in Australia whereby positive cases of select STIs and BBVs are notified to the state and territory health departments. A major limitation of passive surveillance is that it only collects information on positive cases and notifications are heavily dependent on testing patterns. Denominator testing data are important in the interpretation of notifications.

**Objective:**

The aim of this study is to establish a national pathology laboratory surveillance system, part of a larger national sentinel surveillance system called ACCESS (the Australian Collaboration for Coordinated Enhanced Sentinel Surveillance). ACCESS is designed to utilize denominator testing data to understand trends in case reporting and monitor the uptake and outcomes of testing for STIs and BBVs.

**Methods:**

ACCESS involves a range of clinical sites and pathology laboratories, each with a separate method of recruitment, data extraction, and data processing. This paper includes pathology laboratory sites only. First established in 2007 for chlamydia only, ACCESS expanded in 2012 to capture all diagnostic and clinical monitoring tests for STIs and BBVs, initially from pathology laboratories in New South Wales and Victoria, Australia, to at least one public and one private pathology laboratory in all Australian states and territories in 2016. The pathology laboratory sentinel surveillance system incorporates a longitudinal cohort design whereby all diagnostic and clinical monitoring tests for STIs and BBVs are collated from participating pathology laboratories in a line-listed format. An anonymous, unique identifier will be created to link patient data within and between participating pathology laboratory databases and to clinical services databases. Using electronically extracted, line-listed data, several indicators for each STI and BBV can be calculated, including the number of tests, unique number of individuals tested and retested, test yield, positivity, and incidence.

**Results:**

To date, over 20 million STI and BBV laboratory test records have been extracted for analysis for surveillance monitoring nationally. Recruitment of laboratories is ongoing to ensure appropriate coverage for each state and territory; reporting of indicators will occur in 2019 with publication to follow.

**Conclusions:**

The ACCESS pathology laboratory sentinel surveillance network is a unique surveillance system that collects data on diagnostic testing, management, and care for and of STIs and BBVs. It complements the ACCESS clinical network and enhances Australia’s capacity to respond to STIs and BBVs.

**International Registered Report Identifier (IRRID):**

DERR1-10.2196/13625

## Introduction

### Background

The burden of sexually transmitted infections (STIs) and blood-borne viruses (BBVs) compromises quality of life, sexual and reproductive health, and child health, and they can impose a significant financial burden on both the health system and household [1]. STIs—including chlamydia, gonorrhea, and syphilis—and BBVs—including hepatitis B virus (HBV), hepatitis C virus (HCV), and HIV—remain a major public health problem in Australia. Australian notification data revealed that chlamydia and gonorrhea were the second and fourth most notified of all notifiable conditions in Australia in 2016, respectively, and the number of notifications for each has increased steadily since 2000. Infectious syphilis has also increased, with the number of notifications doubling between 2003 and 2017. The number of notifications from HIV has decreased in the last 5 years; however, ongoing study is needed to monitor infections in people who acquire HIV from heterosexual sex and in Aboriginal and Torres Strait Islander people. Chronic HBV infection has remained relatively stable; however, underdiagnosis remains a concern. An increase in HCV notifications was observed in 2015 and 2016, likely in relation to an increase in testing because of the availability of new direct-acting antiviral medications [2,3].

In 2016, the World Health Organization launched a series of global health sector strategies (2016-2021) that outlined goals for ending STIs [1], HIV [4], and viral hepatitis [5] by 2030. Surveillance is recognized as an essential component to measure progress made in each strategy. In many high-income countries, STI and BBV surveillance has traditionally encompassed passive surveillance (ie, case reporting). Passive surveillance is the principal method of STI and BBV surveillance in Australia whereby all states and territories have legislated the notification of all positive cases of select STIs and BBVs from clinicians and pathology laboratories to state and territory health departments [6]. The benefits and limitations of passive surveillance have been well documented; passive surveillance is relatively inexpensive, can cover large geographical areas, and is able to detect disease outbreaks; however, it only collects information on positive cases and notifications are heavily dependent on testing patterns [7,8]. Testing patterns are, in turn, dependent on recommendations and guidance, for example, in Australia, higher risk gay and bisexual men who have sex with men are recommended to be offered STI and BBV testing up to four times per year [9] and pregnant women are recommended to be screened for HBV and HIV, at a minimum [3,9]. Denominator testing data (ie, the total volume of tests conducted) are important in the interpretation of notifications but passive surveillance traditionally does not collect these data. In addition, STI and BBV infections are frequently asymptomatic and, therefore, diagnosis rates will underrepresent true incidence and prevalence.

Several high-income countries, including the United States and the United Kingdom, have implemented pathology laboratory surveillance systems to monitor STIs and BBVs [10-15]. Pathology laboratory surveillance, a form of sentinel surveillance, is used to complement passive surveillance whereby data are collected from a limited number of reporting (*sentinel*) sites. Sentinel surveillance systems are not intended to capture all testing or diagnostic data; rather, they aim to provide a representative sample of those at risk of infection [16]. Sentinel surveillance can be used to measure the burden of disease for infections that are not notifiable and to monitor priority populations in greater detail. Furthermore, when line-listed data are collected for individuals in a comprehensive sentinel surveillance system, a range of additional and more complex analyses are enabled. These include monitoring adherence to STI or BBV prevention and management guidelines (such as frequency of testing and retesting), outcomes of treatment and vaccination (such as HCV cure, HIV viral suppression, and HBV immunity), and other epidemiological outcomes such as incidence, which can be calculated using repeat testing methods.

### Objective

Recognizing the importance of testing denominator data for surveillance, this study aims to establish a national pathology laboratory surveillance system, part of a larger national sentinel surveillance system called ACCESS (the Australian Collaboration for Coordinated Enhanced Sentinel Surveillance). ACCESS was originally implemented in 2007 as a chlamydia-only system [17] and it demonstrated utility to monitor chlamydia testing and positivity in priority populations and sentinel health services and laboratories across Australia [17-22]. This paper describes the purpose, design, and potential of pathology laboratory surveillance in ACCESS.

## Methods

### Study Design and Aims

ACCESS involves a range of clinical sites (including sexual health clinics, general practice clinics, drug and alcohol services, community-led testing services, and hospital outpatient clinics) and pathology laboratories, each with a separate method of recruitment, data extraction, and data processing; this paper includes pathology laboratory sites only, and the establishment of clinical sites has been previously described [23]. ACCESS expanded in 2012 to capture all diagnostic and clinical monitoring tests for STIs and BBVs, initially from pathology laboratories in the 2 largest states in Australia (New South Wales and Victoria), and in 2016 expanded to capture data from at least one public and one private pathology laboratory in all Australian states and territories. The pathology laboratory sentinel surveillance system incorporates a longitudinal cohort design whereby all diagnostic and clinical monitoring tests for STIs and BBVs are collated from participating pathology laboratories in a line-listed format.

The aim of ACCESS is to underpin Australia’s strategic response to STIs and BBVs by maintaining a surveillance system to monitor the testing, diagnosis, and management of these infections and evaluate the impact of relevant health interventions.

### Setting

Australia has a system of both public and private pathology laboratory services with the organization of public pathology laboratories varying across jurisdictions. For example, in New South Wales, services are organized around hospital networks, whereas in Victoria, pathology laboratories operate through individual public hospitals. Public pathology laboratories also provide pathology services to some community-based services, including state and territory-funded sexual health services. Free access to public pathology services is jointly funded by the Australian state and territory governments principally through the National Healthcare Agreement. Private pathology services are the main provider of community-based pathology services. Private pathology laboratories operate specimen collection services across urban, rural, and remote parts of Australia and provide pathology services in several private hospitals on a contracted basis. Private pathology services in the community and in private hospitals are subsidized by the Australian Government through the Medicare Benefits Schedule [24].

### Eligibility and Recruitment

As noted above, ACCESS was initially restricted to New South Wales and Victoria—Australia’s most populous states—representing 32% and 26%, respectively, of Australia’s total population of 23,401,892 in 2016 [25], but with the expansion in 2016, all public and private pathology laboratories are currently eligible to participate in ACCESS if they conduct STI and BBV testing, with ongoing recruitment of pathology laboratories at this time.

**Figure 1:**
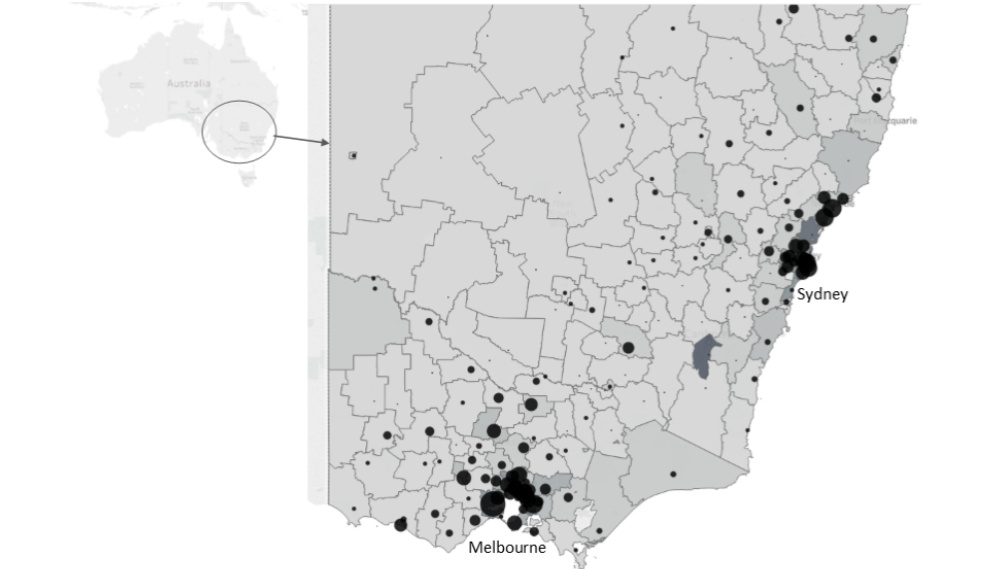
Location of pathology laboratory collection centres in Victoria and New South Wales by Local Government Area, 2016. Note: Postcodes have been converted to Australian Local Government Areas for mapping purposes. Local Government Areas are shaded according to the size of the estimated resident population; darker colours have larger population sizes. Marker for laboratory location is proportional to the number of laboratories located in each local government area.

Information on the size and scope of pathology laboratories is maintained by NRL (formerly known as the National Serology Reference Pathology laboratory, a not-for-profit scientific organization that exists to improve the quality of pathology laboratories testing for infectious diseases). Sites will be selected based on their size, geographical coverage, and clinics that they serviced and will be contacted directly by ACCESS staff and invited to participate. In the initial expansion between 2012 and 2016, a total of 15 pathology laboratories were recruited, including 7 from New South Wales (4 public and 3 private) and 8 from Victoria (6 public and 2 private) representing 41% of the 37 pathology laboratories in those 2 jurisdictions that conduct and report HIV testing data via a quality assurance system [26], and include 971 collection centers (Figure 1). Following the expansion of ACCESS in 2016, 4 more pathology laboratories have been recruited from 3 jurisdictions, including the Australian Capital Territory, Queensland, and Tasmania, resulting in a total of 15 pathology laboratory services. Recruitment is ongoing in the remaining Australian states and territories. Pathology laboratories will receive an establishment payment of Aus $2000 and an annual payment of Aus $500 in subsequent years. This payment is intended to reimburse time and equipment required to establish and maintain data extraction.

### Coordination and Governance

ACCESS is a collaboration among the Burnet Institute, Kirby Institute, and NRL. Table 1 provides the description of data extracted.

### Data Extraction and Linkage

Filters will be used to extract tests related to the diagnosis or management of STIs (chlamydia, gonorrhea, and syphilis) and BBVs (HIV, HBV, and HCV). The extraction and interpretation of tests will be guided by several policies and guidelines, including the Australian STI Management Guidelines for Use in Primary Care [9], the National HIV Testing Policy [27], the National HCV Testing Policy [28], and the National HBV Testing Policy [29]. A list of variables required will be provided to each pathology laboratory to allow pathology laboratory database engineers to map the required variables from their individual pathology laboratory database (see Table 1). Laboratories throughout Australia use assays from different manufacturers and generally have internal selection processes for the selection of markers specific to their laboratory. Some networks of laboratories, especially in the private sector, have uniform selection of assay manufacturer or marker selection, but uniform selection is not universal and cannot be assumed.

Test data comprising 100 lines of data will be extracted initially and provided to the ACCESS team to ensure data items are mapped correctly. Deidentified, encrypted pathology laboratory testing data will then be extracted using GRHANITE data extraction software, developed by the Health and Biomedical Informatics Centre’s GRHANITE Health Informatics Unit at the University of Melbourne [30]. Following installation of GRHANITE at each pathology laboratory, retrospective data from January 1, 2009, to the date of installation will be extracted. Subsequent data extraction will be scheduled monthly, with the previous month’s data collected at each extraction. The accuracy of GRHANITE as a mechanism for data extraction is high; a 2012 review of extracted chlamydia test data from primary health clinics by GRHANITE reported that all chlamydia tests were identified and extracted [31].

**Table 1:** Description of data extracted.

Domain	Data collected
Pathology laboratory	Pathology laboratory name and location
Patient	Generated unique patient identification number; Sex (Male/Female); Patient postcode; Year of birth; Patient ID number at requesting clinic
Request	Date of consultation; Requesting clinician provider number; Clinic name; Clinic postcode
Testing	Date of test; Specimen identification number at pathology laboratory; Pathology laboratory of origin; Test code; Specimen type; Specimen site; Test result; Notes

For each test, information will be extracted on patient demographics (patient sex, patient age at test, and patient postcode), request details (requesting clinician, clinician provider number, and date of test request), and testing details (including test name, specimen site, result, and date of test; Table 1).

GRHANITE will create a unique, encrypted, and nonreversible patient identifier for individuals within each pathology laboratory, using the available patient demographic data. To link the same individual, both within and between pathology laboratories, GRHANITE software will also generate statistical linkage keys (encrypted and nonreversible hash values) using a range of combinations including surname, first/given name, sex, date of birth, Medicare number, and postcode. The statistical linkage key will be created before data extraction and no identifiable data will be extracted. Using the GRHANITE linkage tool software, matches between statistical linkage keys will be identified. On the basis of these matches, a new linkage identification number will be assigned to each unique patient identifier to allow the identification of tests from the same individual within and between pathology laboratories.

### Data Transfer and Storage

Encrypted data files will be received on the Burnet Institute databank server and imported into a Microsoft Structured Query Language Server database. Both the encrypted files and database will be located within a secure password-protected network on a secure server managed by GRHANITE developers and the ACCESS team. Data will be processed within the database using various statistical software packages.

### Data Management

The format and shape of the data to be extracted from each pathology laboratory varies, for example, some pathology laboratories store data as a wide data file including an individual’s historical testing data, whereas some store data as a long data file with each test and accompanying result on a separate line. Data management personnel will map and convert data to a consistent format and shape and merge the data files. Each test type (see Table 2) will then be mapped to a disease group, and data will be separated into disease-specific datasets for cleaning and management. Raw data will be converted to qualitative results where possible (negative, positive, and indeterminate). When quantitative results are provided for qualitative tests, pathology laboratories will be contacted to provide reference ranges. The dataset will then be restructured to represent 1 line per testing event to enable accurate test interpretation using case definitions. Duplicates and missing test result data will be excluded.

### Analyses

Several important epidemiological indicators will be calculated utilizing data from pathology laboratory sites and are described in Table 2. Where appropriate, indicators will be stratified by sex (male and female) and age at time of test. Individuals with missing key demographic information (ie, sex or age) will be excluded from all analyses. Tests conducted within 7 days of each other will be considered a part of the same clinical encounter and, therefore, collapsed into the earliest visit date.

Where appropriate, pathology laboratory data will be appended to data collected from clinical services participating in ACCESS. The same indicators listed above will also be calculated; however, the expanded dataset will provide a more comprehensive description of STI and BBV testing, diagnosis, and management. Where possible, demographic data collected in clinical sites (such as sexual orientation or behavior and ethnicity, for example) will be linked to patients in pathology laboratory data to improve the characterization of individuals.

**Table:** 

Indicator	Sexually transmitted disease / Blood-borne virus	Description
Total number of tests	All	The total number of tests conducted
Unique number of individuals tested	All	The total number of unique individuals that received at least one test
Test yield	All	The total number of positive results, divided by the total number of tests, expressed as a percentage
Proportion positive	All	The total number of unique individuals with a positive result, divided by the total number of unique individuals tested, expressed as a percentage
Repeat test for test of cure	Chlamydia	Test for chlamydia within six weeks of a previous positive test
Repeat test for reinfection	Chlamydia	Test for chlamydia at least six weeks after a previous positive test
Incidence	All	Among patients with at least two tests and whose first test result was negative, the number of unique incident cases divided by the total person time contributed by unique individuals who tested
Immunity	Hepatitis B virus	An anti- hepatitis B surface antibody titer measured as being >10mIU/L

### External Use of Data

External researchers will be able to make data requests to access and utilize data collected in ACCESS. Applications will be considered by a central ACCESS coordinating committee and will require appropriate ethical approvals.

### Ethical Approval

Ethical approval was provided by the Alfred Hospital Ethics Committee (Project No. 90/12 and Project No. 248/17). Individual consent is not collected in this study, in line with national standards [32].

## Results

To date, over 20 million STI and BBV laboratory test records have been extracted for analysis for surveillance monitoring nationally. Recruitment of laboratories is ongoing to ensure appropriate coverage for each state and territory; reporting of indicators will occur in 2019 with publication to follow.

## Discussion

ACCESS is a novel and innovative surveillance system that provides important data that can be used to understand trends in case reporting and monitoring the uptake and outcomes of testing for STIs and BBVs in Australia.

### Strengths

There are several key strengths to the ACCESS pathology laboratory sentinel surveillance system. First, the use of specialized software enables the anonymous linkage of individuals between pathology laboratories and clinical sites. We are unaware of any similar STI or BBV surveillance system that has the capacity for such broad data linkage internationally. The capacity for data linkage across services and service types is particularly important, given that people may access multiple services for testing and management of STIs and BBVs [33,34]. The extent of use and the crossover of attendance at multiple services and service types is not known in Australia, and analysis of service-specific data is likely to underascertain service utilization and disease incidence in many risk groups. Second, some STIs—notably chlamydia—are most commonly diagnosed in general primary care services, undermining the potential to recruit a representative sample of sentinel clinical sites. Coverage via pathology laboratory data is a more effective and efficient way of monitoring these infections. Third, data collected in pathology laboratory sites have broad utility in monitoring disease incidence and prevalence as well as monitoring cascades of care by tracking individuals’ progression from diagnosis through ongoing care. For example, follow-up of an individual with an HIV diagnosis can be observed using the HIV viral load test records that follow. If the individual’s viral load reaches below a particular threshold, it is an indication that the individual is on treatment and is no longer able to transmit HIV. Data can also be linked to additional datasets in clinical services that provide enhanced information on service utilization and membership of priority populations. Finally, because of flexibility of the data extraction system, it is possible to add additional tests to the pathology laboratory extraction with minimal resources, if this is required by the Departments of Health in the future.

### Limitations

Some limitations to the system should be considered. First, we are unable to determine if the first identified case of a chronic infection (such as HIV, HBV, or HCV) is the date of diagnosis, as an individual may have first tested positive before 2009 (the earliest date of data extraction), and thus a positive test may not represent a new diagnosis. However, a pilot is underway to link the ACCESS pathology laboratory data to Australian BBV passive surveillance systems to help resolve this issue. Second, a significant amount of STI and BBV testing in Australia occurs in private pathology laboratories and not all private pathology laboratories currently participate in ACCESS. However, sentinel surveillance systems are not intended to capture all testing or positivity data, rather they should provide a representative sample of the population of interest. The representativeness of the system has not been established; however, geographical visualization of pathology laboratory collection sites (see Figure 1) suggests that there is reasonable coverage across urban and regional areas of Victoria and New South Wales. Furthermore, the anticipated linkage to passive surveillance data will help assess the representativeness of those being diagnosed with an STI or BBV. Finally, other than chlamydia, STIs and BBVs are concentrated in priority populations, defined by sexual identify, ethnicity, or risk practices (such as injecting drug use)—information that is not routinely collected by pathology laboratories. ACCESS is currently exploring ways to identify these populations in pathology laboratory data. For example, ACCESS collaborators recently analyzed data from a semiautomated sentinel surveillance system that preceded ACCESS in Victoria [35] to explore the potential of using testing pattern data to identify gay and bisexual men who have sex with men in STI surveillance systems. It was found that if an individual was identified as ever having an anorectal swab (for chlamydia or gonorrhea testing), it was a highly predictive and valid marker of gay and bisexual men who have sex with men attending sexual health clinics [36]. Additional predictive algorithms to identify priority populations will be investigated and validated in ACCESS pathology laboratory and clinical sites. For example, pregnant women receiving antenatal screening could be identified through concurrent rubella, syphilis, and HBV testing and people from culturally and linguistically diverse communities could be identified through the use of electronic ethnicity classification software [13,14].

### Conclusions

The ACCESS pathology laboratory sentinel surveillance network is a unique surveillance system that collects data on regular diagnostic testing and linkages to and retention in care. Its initial implementation as a chlamydia surveillance system provided a platform from which the system can be expanded. This system can inform the development of surveillance systems globally to measure progress made toward reaching both local and global targets for reducing the impact of STIs and BBVs.

## Abbreviations

ACCESS: Australian Collaboration for Coordinated Enhanced Sentinel Surveillance
BBV: blood-borne virus
HBV: hepatitis B virus
HCV: hepatitis C virus
STI: sexually transmitted infection
